# Design of a 3D Aggregation Backbone Network-on-Chip Architecture for High-Speed Data Acquisition Systems

**DOI:** 10.3390/mi17101119

**Published:** 2026-09-24

**Authors:** Yunhui Deng, Chuanpei Xu, Wei Mo, Chunting Wan

**Affiliations:** 1 School of Electronic Engineering and Automation, Guilin University of Electronic Technology, Guilin 541004, China; 20081101006@mails.guet.edu.cn (Y.D.); wmo@guet.edu.cn (W.M.); wallct@guet.edu.cn (C.W.); 2 Guangxi Key Laboratory of Automatic Detecting Technology and Instruments, Guilin 541004, China

**Keywords:** network-on-chip, aggregation backbone architecture, deterministic routing, high-speed data acquisition system

## Abstract

Time-interleaved high-speed data acquisition (HS-DAQ) systems based on three-dimensional network-on-chip (3D NoC) architectures require efficient transmission of multi-channel sampled data. However, existing general-purpose NoC architectures are not well suited for the deterministic multi-source aggregation communication pattern of HS-DAQ systems, which may result in increased long-distance transmission overhead and localized traffic congestion. To address these issues, this paper proposes a 3D aggregation backbone network-on-chip architecture (3D AB-NoC) for HS-DAQ systems. This architecture is based on a 3D Mesh structure and enables the rapid transmission of long-distance aggregated data by establishing coarse-grained aggregation backbone subnets between defined aggregation routing nodes. Furthermore, a Backbone-Boosted XYZ (BB-XYZ) routing algorithm is proposed, which selects the next hop based on the remaining transmission distance of both the basic mesh path and the backbone path, thereby achieving coordinated routing for both local transmission and long-distance aggregation. Simulation results based on the Access Noxim platform demonstrate that the 3D AB-NoC maintains stable basic communication performance under general communication scenarios. In HS-DAQ communication scenarios, the proposed architecture reduces average latency by up to approximately 44%, improves throughput by approximately 29.3%, and significantly suppresses the growth of maximum latency under high-load conditions.

## 1. Introduction

To improve the overall sampling bandwidth of data acquisition systems, the time-interleaved analog-to-digital converter (TIADC) architecture has been widely adopted in high-speed data acquisition systems. By employing multiple sub-ADCs operating in parallel, TIADC enables high-speed data sampling [1]. In large-scale TIADC systems, multiple acquisition nodes continuously generate high-frequency sampled data, which need to be transmitted in real time to backend storage nodes and transmission nodes for data reconstruction and processing. This requirement imposes higher demands on the transmission performance and scalability of on-chip communication architectures [2]. However, with the continuous increase in the number of sampling channels and data throughput requirements of HS-DAQ systems, conventional on-chip interconnection methods are becoming insufficient for multi-channel real-time data transmission. Therefore, HS-DAQ systems based on three-dimensional on-chip networks have emerged as a highly promising solution for enhancing the parallel communication capabilities of such systems.

In NoC-based HS-DAQ systems, multiple acquisition nodes transmit sampled data to corresponding storage nodes according to predefined data flow relationships, and multiple storage nodes further aggregate the data toward a unified transmission node. Since acquisition tasks usually exhibit fixed data generation rates and deterministic transmission directions, the communication process of the system presents characteristics of multi-source aggregation, unidirectional transmission, and stable traffic directions. Consequently, this imposes specific requirements on path organization, traffic distribution, and transmission efficiency of the NoC architecture.

Three-dimensional NoC architectures employ through-silicon vias (TSVs) to enable high-speed inter-layer communication [3]. Compared with 2D NoC, they offer clear advantages in terms of network diameter, communication parallelism, and integration density, and have therefore been widely adopted in high-performance system-on-chip designs. Among various 3D NoC topologies, the 3D Mesh has become one of the most commonly used architectures because of its regular structure, low implementation complexity, and good scalability [4]. However, the 3D Mesh interconnect nodes based on adjacency rules, and its hop-by-hop transmission mechanism is not well suited to the deterministic, unidirectional, and progressively aggregating traffic pattern of time-interleaved HS-DAQ systems. As the network size and data acquisition rate increase, sampled data generated by acquisition nodes located far from their destinations must traverse longer multi-hop paths, resulting in increased transmission latency and forwarding overhead. Therefore, an NoC architecture better suited to time-interleaved HS-DAQ systems is required.

To address the above issues, we propose a 3D Aggregation Backbone Network-on-Chip architecture for HS-DAQ systems. Building upon traditional 3D Mesh NoC, this architecture introduces a coarse-grained aggregation backbone subnet. By periodically deploying aggregation backbone routers and adding backbone links, it constructs a hierarchical communication structure optimized for long-distance aggregation and transmission. Meanwhile, a backbone-assisted routing strategy is further designed to dynamically select between the conventional Mesh path and the backbone-subnet path according to different communication path lengths. This enables hierarchical transmission of long-distance aggregation traffic and local communication traffic.

Different from existing long-range-link or hierarchical NoC architectures that mainly optimize general communication efficiency, this work focuses on the deterministic many-to-few aggregation characteristics of HS-DAQ systems. The main idea of this work is to establish an application-oriented communication architecture by matching the network topology and routing mechanism with the inherent aggregation behavior of acquisition data streams.

The main contributions of this work are summarized as follows:

(1) To address the poor compatibility between deterministic multi-source aggregation traffic patterns in NoC-based high-speed data acquisition systems and 3D Mesh NoC architectures, a 3D AB-NoC architecture is proposed. This provides an efficient transmission path for long-distance data acquisition;

(2) A deterministic transmission mechanism incorporating the backbone subnet is designed, including node deployment and a Backbone-Boosted XYZ routing algorithm. The proposed mechanism enables packets to select either Mesh paths or backbone paths according to path cost, thereby reducing aggregation transmission overhead and alleviating localized traffic concentration.

The remainder of this paper is organized as follows. Section 2 reviews the related work and presents the motivation for this study. Section 3 introduces the communication model and data flow characteristics of HS-DAQ systems. Section 4 then provides a detailed description of the proposed 3D AB-NoC architecture, the HS-DAQ system model based on this architecture, the Backbone-Boosted XYZ routing algorithm, and their structural characteristics. Section 5 presents the simulation results and experimental analysis. Finally, Section 6 concludes the paper.

## 2. Related Work

In recent years, extensive research efforts have been devoted to improving the communication performance of network-on-chip (NoC) systems, focusing on network topology design, routing algorithms, mapping strategies, and resource allocation. With regard to topologies, researchers have proposed a variety of interconnection architectures. S. Kumar et al. [5] proposed the most classic m × n two-dimensional mesh architecture, which features a large bisection width. However, this mesh architecture suffers from a large network diameter, which can easily lead to high communication latency. Therefore, several studies have attempted to reduce the average hop count and improve data transmission efficiency by introducing long-range links, Small-World structures, and hierarchical interconnection mechanisms. Balfour J. D. et al. [6] proposed the CMesh architecture. In the CMesh network, each router is connected to four IP cores, which effectively reduces the network scale and consequently decreases the average hop count. Varanasi L. K. et al. [7] proposed a diagonal network topology named DiamondMesh. By introducing diagonal links into the conventional mesh topology, DiamondMesh reduces the network diameter and thereby decreases long-distance communication latency. A. Alagarsamy et al. [8] proposed a 2D NoC topology called Four Regular Dense Spidergon (FRDS). This topology reduces the average network hop count through a specially designed ring-star hybrid structure. V. L. Kiranmai et al. [9] proposed a Hybrid Hexagonal Star (HS) topology that combines hexagonal and star structures. The HS topology reduces link lengths through non-Manhattan routing and the high adjacency of the hexagonal structure, while improving connectivity and scalability. C. Li et al. [10] proposed a highly scalable NoC architecture, namely TriBA-mNoC, for triplet-based many-core systems. This architecture is based on a ternary tree topology and significantly improves memory access performance in large-scale many-core systems. Meanwhile, the designed shared hierarchical grouping structure effectively addresses the non-uniform memory access (NUMA) problem. A. Bose et al. [11] proposed a three-dimensional butterfly fat-tree (3D BFT)-based NoC architecture. By introducing a zone-based routing strategy and boundary router design, this architecture effectively alleviates the upward traffic congestion and long interconnection wire issues encountered by conventional BFT architectures in large-scale networks.

Meanwhile, some studies have focused on optimizing the distribution of through-silicon via (TSV) resources for vertical communication in 3D NoC. R. G. Kunthara et al. [12] proposed a three-dimensional multilayer topology based on interleaved edge routers. By utilizing the available ports of edge routers in Mesh or CMesh networks to establish partially connected TSV channels, the proposed topology reduces both the number of TSVs and the associated area overhead. Other research efforts [13,14] have proposed a series of TSV placement optimization and congestion-aware scheduling strategies for partially connected 3D NoC (3D PC-NoC). These approaches include optimizing vertical-link placement according to traffic distribution and dynamically adjusting inter-layer communication paths at runtime through an elevator assignment mechanism [15].

In summary, existing NoC topology optimization approaches mainly improve overall network communication efficiency by modifying node connectivity, introducing auxiliary links, or constructing hierarchical interconnection structures. These approaches can effectively reduce the average path length under general-purpose traffic patterns and improve the scalability of large-scale networks. However, most existing architectures are primarily designed for relatively uniform or random inter-node communication patterns, while the specific communication characteristics of application-oriented systems have not been sufficiently considered.

To further clarify the differences between existing long-range or hierarchical NoC architectures and the proposed architecture, a comparison is summarized in Table 1. The comparison considers topology characteristics, additional connectivity, router requirements, routing mechanisms, and target traffic patterns. Therefore, targeting the aggregation-oriented communication requirements of HS-DAQ systems, we propose an aggregation backbone network-on-chip architecture.

## 3. Communication Characteristics of HS-DAQ Systems

HS-DAQ systems employ time-interleaving techniques to achieve high-speed sampling. The multiple acquisition nodes in the system are typically implemented using analog-to-digital converters (ADC) of the same type and with identical sampling rates. Therefore, the data injected into the NoC by each acquisition node have the same size and transmission rate, while the sampling start time differs among the nodes [16]. Figure 1 illustrates the sampling timing of four time-interleaved ADCs.

In the HS-DAQ system, NoC router nodes can be classified into four functional types according to the IP cores: acquisition nodes, storage nodes, transmission nodes, and control nodes. The communication model among these functional nodes is shown in Figure 2. The control node is mainly responsible for system operation control and transmits control information before the start or at the end of a system task. Therefore, it does not participate in the main transmission process of sampled data. The acquisition nodes receive sampled data from the ADC and continuously transmit data packets to designated storage nodes. After the storage nodes complete data reconstruction and buffering, the received data are further forwarded to the transmission node for final reconstruction and output.

The communication relationships in high-speed data acquisition systems are generally determined during the system design stage. Each acquisition node communicates only with a designated storage node, while the storage nodes further transfer data to the corresponding transmission node [17]. As a result, the overall data flow presents the following communication characteristics:

1. Deterministic Data Flow Transmission: NoC-based HS-DAQ systems typically establish fixed data transmission relationships according to pre-configured data acquisition tasks. After the acquisition task starts, each acquisition node transmits sampled data to its corresponding storage node according to the predefined sampling channel and data flow direction;
2. Data Aggregation Communication: In systems, multiple acquisition nodes transmit data to the same storage node for aggregation, and multiple storage nodes further send the stored data to the transmission node. Consequently, data flows gradually converge toward a small number of destination nodes, forming a typical aggregation communication pattern;
3. Directional Communication: The data transmission direction in HS-DAQ systems exhibits strong unidirectional characteristics. Most communication behaviors follow fixed paths among acquisition nodes, storage nodes, and transmission nodes. The data flows have clear transmission directions, while large-scale random access to arbitrary destination nodes will not occur.

When an HS-DAQ system is mapped to a 3D Mesh NoC, the acquisition nodes are distributed across different network layers and plane regions. As the scale of the network expands, the length of the data transmission paths increases, which not only increases the basic transmission latency but also introduces additional router forwarding and link switching overhead.

Furthermore, the major communication traffic in the network exhibits a spatial distribution pattern where data flows gradually converge from distributed acquisition regions toward localized aggregation regions. The 3D Mesh architectures mainly rely on hop-by-hop transmission between neighboring nodes. When handling cross-region aggregation traffic with fixed transmission directions, longer communication paths and localized traffic concentration become more significant.

## 4. Proposed 3D Aggregation Backbone NoC Architecture

### 4.1. Architecture Design of 3D AB-NoC

To address the high transmission overhead of long-distance aggregation traffic in 3D Mesh architectures and the localized load concentration caused by data aggregation, a 3D AB-NoC architecture for HS-DAQ systems based on a regular 3D Mesh structure is proposed. The core idea of this architecture is to define a small number of key nodes as aggregation points within the network while preserving the regularity of the overall mesh topology. Coarse-grained backbone connections are then established between these aggregation nodes, thereby forming a hierarchical communication structure capable of supporting efficient long-distance transmission. This approach provides efficient data transmission channels for major communication paths without significantly increasing structural complexity. The 3D AB-NoC architecture consists of the following three basic components.

1. Regular nodes and links: Regular nodes and links retain the conventional connectivity of a Mesh NoC and are responsible for receiving and forwarding data within local regions. They serve as the basic communication units of the aggregation backbone architecture.
2. Aggregation nodes: Aggregation nodes are key nodes selected according to predefined deployment rules and are mainly responsible for cross-region or long-distance communication. Compared with regular router nodes, aggregation nodes establish long-range interconnections through additional backbone links.
3. Coarse-grained backbone links: Coarse-grained backbone links connect different aggregation nodes and form a backbone subnet covering the network. These links are sparsely deployed and are primarily intended to shorten long-distance communication paths and enable efficient cross-region data transmission.

Through the above components, the overall network logically forms a hierarchical structure consisting of the basic 3D Mesh and an aggregation-backbone subnet. Regular routers retain the conventional nearest-neighbor Mesh connections, whereas selected aggregation routers additionally provide long-range backbone connectivity for persistent aggregation traffic.

To ensure deterministic and reproducible deployment, the aggregation routers are placed periodically according to a fixed spacing parameter s, rather than being selected according to a specific application mapping. For an $N_{x}\times N_{y}\times N_{z}$ network, a router located at $\left(x,y,z\right)$ is selected as an aggregation router when both x and y are integer multiples of s. Therefore, the aggregation-router set is completely determined by the network dimensions and the spacing parameter. The definition and number of aggregation nodes in the NoC can be expressed by Equations (1)–(4):

(1) $$A=\left\{\left.\left(x,y,z\right)\right|x\;\mathrm{mod}\;s=0,y\;\mathrm{mod}\;s=0\right\}$$

(2) $$N_{A}=N_{z}\times N_{Ax}\times N_{Ay}$$

(3) $$N_{Ax}=\left\lfloor \left(N_{x}-1\right)/s\right\rfloor +1$$

(4) $$N_{Ay}=\left\lfloor \left(N_{y}-1\right)/s\right\rfloor +1$$

where A is the set of aggregation routers; $N_{A}$ is the number of aggregation nodes in the NoC; and $N_{x}$, $N_{y}$, and $N_{z}$ are the dimensions of the network in the x, y, and z directions, respectively.

Within each horizontal layer, neighboring aggregation routers are connected along the x and y directions to form the aggregation backbone. Inter-layer communication is provided by the TSV links of the underlying 3D Mesh. The “3D AB-NoC” refers to the overall three-dimensional NoC architecture, while the dedicated aggregation backbone itself is constructed within each horizontal layer.

Accordingly, the number of aggregation routers and backbone links increases deterministically with the network dimensions and the selected spacing. A smaller value of s results in a denser backbone with more aggregation routers and additional links, whereas a larger value of s reduces the backbone density but increases the physical span of each backbone link. The relationship between the number of additional links added between aggregation nodes and the network scale is as follows:

(5) $$N_{backbone}=N_{z}\times \left[\left(N_{Ax}-1\right)\times N_{Ay}+N_{Ax}\times \left(N_{Ay}-1\right)\right]$$

A regular router retains the original Mesh ports. An aggregation router additionally contains backbone ports corresponding to its valid neighboring aggregation routers. Let $d_{B}$ denote the number of backbone neighbors of an aggregation router. Its router degree can therefore be expressed as $p_{A}=7+d_{B}$.

Figure 3 illustrates a 4 × 4 × 3 3D AB-NoC architecture. Each layer consists of a basic Mesh network (represented by the dark-gray links) and a coarse-grained backbone Mesh subnet (represented by the red links). Communication between different layers is implemented through vertical TSV channels.

### 4.2. Functional Node Deployment and HS-DAQ System Model

In a 3D NoC, the spatial distribution of functional nodes directly affects communication efficiency and system thermal distribution. Therefore, different types of functional nodes should be reasonably deployed according to the communication characteristics of high-speed data acquisition systems and the physical characteristics of 3D integrated circuits.

Storage nodes and transmission nodes usually need to be connected with peripheral interfaces or memory controllers of the system. Compared with acquisition nodes, storage nodes and transmission nodes are responsible for larger-scale data transmission and processing. Since the bottom layer of a NoC generally provides better heat dissipation conditions and more convenient connections to external I/O interfaces, deploying storage nodes and transmission nodes on the bottom layer of the NoC can reduce the overall thermal load and improve system reliability. To reduce transmission path length and improve data aggregation efficiency, storage nodes are preferentially deployed on aggregation nodes. In addition, storage nodes perform buffering and data-rate matching between acquisition data and backend output interfaces, and the stored data generally need to be further sent to transmission nodes. Consequently, placing storage nodes close to transmission nodes can reduce additional on-chip forwarding overhead and link occupation during the stored data output process.

Acquisition nodes are responsible for data sampling, and their primary communication task is to transmit sampled data to storage nodes. In 3D NoC, vertical TSV channels provide shorter communication distances and lower transmission latency. Therefore, acquisition nodes are distributed on network layers other than the bottom layer and communicate with bottom-layer storage nodes through vertical channels. This deployment enables acquisition nodes to effectively utilize vertical communication channels and improve data transmission efficiency.

Based on the above functional node deployment strategy, a high-speed data acquisition system consisting of 24 acquisition nodes, 3 storage nodes, 1 transmission node, and 1 control node is considered as an example. The corresponding system model based on the proposed 3D AB- NoC architecture is illustrated in Figure 4.

When the distance between an acquisition node and its target storage node is relatively large, the data can utilize the backbone subnet formed by aggregation nodes for long-distance transmission. After receiving the acquisition data, each storage node reconstructs and buffers the data according to the sampling sequence, and then forwards the stored data toward the transmission node for further aggregation. Finally, the transmission node completes the overall data reconstruction process and outputs the integrated data.

### 4.3. Backbone-Boosted XYZ Routing Algorithm

In 3D Mesh NoC, the XYZ routing algorithm is one of the most commonly adopted deterministic routing algorithms [18]. It gradually routes data from the source node toward the destination node in a fixed dimensional order, thereby ensuring deadlock-free routing with relatively low implementation complexity. In the proposed 3D AB-NoC architecture, however, the introduction of backbone links significantly changes the network connectivity and path distribution characteristics. If the conventional XYZ routing algorithm is directly applied, the newly introduced backbone links cannot be effectively utilized by the routing mechanism.

To address this issue, the conventional XYZ routing rule is extended to form the Backbone-Boosted XYZ (BB-XYZ) routing algorithm. BB-XYZ retains the deterministic X–Y–Z dimensional order of conventional XYZ routing. The packet first resolves the x-coordinate difference, then the y-coordinate difference, and finally the z-coordinate difference. Backbone links are used only in the horizontal x and y dimensions, whereas inter-layer transmission continues to use conventional TSV channels.

Let the current router be $C=\left(x_{c},y_{c},z_{c}\right)$ and the destination router be $D=\left(x_{D},y_{D},z_{D}\right)$. The remaining coordinate differences are defined as $d_{x}=x_{d}-x_{c}$, $d_{y}=y_{d}-y_{c}$, and $d_{z}=z_{d}-z_{c}$. During the x-routing phase, if the current router is an aggregation router and $\left|d_{x}\right|>s$, where s is the backbone spacing, the backbone link in the direction of the destination is preferentially selected. Otherwise, a conventional nearest-neighbor x direction Mesh link is used. After the x-coordinate has been completely resolved, the same rule is applied in the y dimension. The remaining z coordinate difference is resolved through conventional TSV links.

A backbone traversal advances the packet by s router pitches in the active horizontal dimension, whereas a conventional Mesh link advances it by one router pitch. Therefore, a backbone link is selected only when the remaining distance is sufficient for a complete backbone traversal without passing the destination. The BB-XYZ algorithm is shown in Algorithm 1.
**Algorithm 1:** BB-XYZ routing algorithmInput: Current router $C=\left(x_{c},y_{c},z_{c}\right)$, destination router $D=\left(x_{D},y_{D},z_{D}\right)$,   backbone spacing sOutput: Output direction dir1: $d_{x}=x_{d}-x_{c}$
2: $d_{y}=y_{d}-y_{c}$
3: anchor = $x_{c}\mathrm{mod}s=0$ and $y_{c}\mathrm{mod}s=0$
4: if $d_{x}>0$ then5:  if backbone enabled and anchor and $d_{x}\geq s$ then6:    dir = Backbone_EAST7:  else8:    dir = EAST9:  end if10: else if $d_{x}<0$ then11:  if backbone enabled and anchor and $\left|d_{x}\right|\geq s$ then12:    dir = Backbone_WEST13:  else14:    dir = WEST15:  end if16: else if $d_{y}>0$ then17:  if backbone enabled and anchor and $d_{y}\geq s$ then18:    dir = Backbone_SOUTH19:  else20:    dir = SOUTH21:  end if22: else if $d_{y}<0$ then23:  if backbone enabled and anchor and $\left|d_{y}\right|\geq s$ then24:    dir = Backbone_NORTH25:  else 26:    dir = NORTH27:  end if28: else if $z_{d}>z_{c}$ then29:  dir = DOWN30: else if $z_{d}<z_{c}$ then31:  dir = UP32: else33:  dir = LOCAL34: end if35: return dir

When multiple candidate output ports satisfy the routing condition, BB-XYZ follows the original XYZ dimensional priority, i.e., the x dimension is selected before the y dimension, and the y dimension is selected before the z dimension. Within the same dimension, the backbone channel is selected preferentially because it provides a longer transmission distance than the corresponding Mesh channel. Therefore, the routing decision is deterministic and unique for each source-destination pair. The flowchart of the proposed BB-XYZ routing algorithm is shown in Figure 5.

The introduction of backbone channels does not change the dimensional order of BB-XYZ. During the x routing phase, a packet may use either conventional x direction Mesh channels or x direction backbone channels, but all traversals maintain the same direction toward the destination. Once the destination x coordinate has been reached, the packet enters the y routing phase and cannot return to the x dimension. Likewise, after entering the z routing phase, the packet cannot return to either horizontal dimension. Within a given dimension, transitions between a Mesh channel and a backbone channel are permitted only when both channels have the same dimension and direction, such as EAST–BEAST, WEST–BWEST, NORTH–BNORTH, and SOUTH–BSOUTH. Direction reversal within the active dimension is not permitted. Therefore, no cyclic dependency can be formed across dimensions.

Within each dimension, routing is also monotonic. EAST and BEAST strictly increase the x coordinate, while WEST and BWEST strictly decrease it; the same property holds for the positive and negative y-directions. TSV routing similarly changes the z coordinate monotonically toward the destination. Since BB-XYZ never reverses direction within the active dimension, no intra-dimensional channel cycle can be formed. Consequently, the overall channel-dependency is acyclic, and BB-XYZ is deadlock-free.

### 4.4. Structural Characteristics Analysis

To quantitatively evaluate the proposed 3D AB-NoC architecture, its key structural characteristics are theoretically analyzed and compared. Metrics such as network diameter and average network distance are commonly used to evaluate the structural properties of NoC topologies. In this work, the 3D Mesh architecture is employed as the baseline, and the structural characteristics of the 3D AB-NoC architecture are analyzed under the same network size and number of nodes.

#### 4.4.1. Network Diameter

Network diameter [19] is an important metric for evaluating NoC topology characteristics. It is defined as the maximum shortest-path length between any pair of nodes in the network and can be expressed as

(6) $$D=\underset{u,v\in V}{\max}\;d\left(u,v\right)$$

where V denotes the set of network nodes, and d(u,v) represents the hop count of the shortest path between nodes u and v. Each traversal through a router is counted as one hop, and the hop count from the source node to the destination node is defined as the total number of routers traversed along the path. The network diameter therefore reflects the worst-case communication distance in the network.

Figure 6 compares the network diameters of the proposed 3D AB-NoC architecture and the conventional 3D Mesh architecture under different network sizes. As shown in the figure, the network diameter of the 3D Mesh NoC and 3D AB-NoC increases linearly with network size. In the proposed 3D aggregation backbone NoC, the backbone subnet formed by aggregation routers enables long-distance traffic to be rapidly forwarded through backbone links, thereby reducing the count of transmission hops in the planar dimensions. The growth rate of the network diameter is therefore significantly lower than that of the 3D Mesh architecture. The results demonstrate that the proposed 3D AB-NoC architecture can effectively reduce the maximum communication path length in large-scale networks.

#### 4.4.2. Average Network Distance

Average network distance [20] is used to measure the average length between arbitrary pairs of nodes in a network. A smaller average network distance indicates that fewer hops are required for data transmission, resulting in higher overall communication efficiency. The average network distance can be calculated using Equation (7), where N denotes the number of routers in the network.

(7) $$D_{avg}=\frac{1}{N\left(N-1\right)}\sum_{u,v\in V}d\left(u,v\right)$$

Figure 7 presents the comparison of the average network distance between the 3D Mesh NoC and the 3D AB-NoC under different network sizes. The average network distance of both architectures gradually increases with the expansion of network size. However, for each network size, the average network distance of the 3D AB-NoC is smaller than that of the 3D Mesh NoC. The result indicates that introducing the backbone subnet can effectively reduce the average communication path length between nodes and improve the overall communication efficiency in large-scale NoC systems.

### 4.5. Hardware Overhead Analysis

Although the proposed 3D AB-NoC architecture improves the communication efficiency for HS-DAQ aggregation traffic, it introduces additional hardware resources compared with the conventional 3D Mesh. These additional resources mainly include the aggregation routers and the dedicated backbone links. Therefore, the hardware overhead introduced by the proposed architecture is analyzed in terms of router resources, backbone connectivity, and interconnection requirements.

In the architecture, regular routers preserve the original 3D Mesh structure and do not introduce additional hardware resources. Only the selected aggregation routers are extended with additional backbone ports to support long-range horizontal communication. Therefore, the hardware overhead is proportional to the number of aggregation routers and backbone links rather than the total number of routers in the network.

The proposed architecture introduces dedicated horizontal backbone links between neighboring aggregation routers. According to the backbone construction rule, the number of additional backbone links in the x and y directions can be determined by the number of aggregation routers in each plane.

Compared with the conventional 3D Mesh router, an aggregation router additionally contains four horizontal backbone ports (BEAST, BWEST, BSOUTH, and BNORTH). Therefore, the maximum port count increases from seven ports in a conventional router to eleven ports in an aggregation router. The proposed backbone subnet only introduces additional horizontal communication paths and does not modify the vertical communication mechanism. Therefore, the TSV structure remains identical to that of the conventional 3D Mesh.

The above analysis shows that the proposed 3D AB-NoC achieves communication acceleration by introducing a limited amount of dedicated backbone resources. The additional hardware is selectively applied to aggregation routers rather than the entire network, while the TSV structure and buffer depth remain unchanged. Therefore, the architecture provides a trade-off between additional connectivity resources and reduced communication overhead for aggregation-dominated HS-DAQ traffic.

To further quantify the additional communication resources introduced by the proposed architecture, the 6×6×4 network with s=2, which specific experimental results are presented in Section 5.2.

A conventional 3D Mesh of this size contains 348 links, while the proposed 3D AB-NoC introduces 48 additional backbone links and 36 aggregation routers. The additional backbone links correspond to an approximately 13.8% increase in the link count. Since each backbone link spans two router pitches, the additional horizontal interconnect length is approximately 96 router pitches. Compared with the 240 router-pitch horizontal interconnects of the conventional 3D Mesh, this corresponds to an approximately 40% increase in horizontal wiring length. In addition, the 36 aggregation routers introduce at most four additional backbone ports each, corresponding to an upper-bound increase of approximately 14.3% in the total router-port count.

These additional resources should be considered together with the corresponding communication benefits. Under the high-load HS-DAQ workload, the proposed architecture reduces the average latency by approximately 44.1% and improves throughput by approximately 29.3%. In contrast, under general random traffic, the throughput improvement is only approximately 1.88–2.60% although the same backbone resources are present. This suggests that the performance benefit is related not only to the additional connectivity, but also to the matching between the aggregation-oriented backbone organization and the HS-DAQ traffic pattern.

## 5. Simulation and Results

To evaluate both the baseline performance of the proposed 3D AB-NoC architecture and its performance under high-speed data acquisition workloads, simulation experiments were conducted under two traffic modes: random traffic injection and emulated high-speed data acquisition traffic injection. Different network sizes of the 3D AB-NoC and conventional 3D Mesh NoC were evaluated. The random traffic injection mode was mainly used to assess the basic network performance of 3D AB-NoC under general communication scenarios, serving as a baseline performance evaluation. In contrast, the high-speed data acquisition traffic injection mode was used to evaluate the optimization effectiveness of the proposed architecture under persistent aggregation traffic conditions.

We used Access Noxim v0.3 [21], developed by Kaiyuan Zheng et al., as an on-chip network simulation platform to experimentally evaluate the proposed network architecture. All experiments were conducted on a computer equipped with an Intel i5-7500 processor and 16 GB of memory. The main simulation parameters are summarized in Table 2.

In the proposed 3D AB-NoC architecture, the backbone links are modeled as additional logical communication channels in the Access Noxim. Similar to conventional Mesh links, each backbone traversal is abstracted as one routing hop in the current architecture-level evaluation. The energy consumption of backbone communication is calculated using the same router and link energy model adopted by the simulator. This abstraction is intended to evaluate the communication-level benefits of the proposed topology and routing mechanism.

The current Access Noxim implementation abstracts each backbone traversal as one logical routing hop. However, a backbone link may span multiple router pitches and therefore should not be regarded as physically identical to a conventional nearest-neighbor Mesh link. To further account for this difference, a simplified wire-length-dependent delay and energy estimation is introduced.

Let $D_{r}$ denote the delay associated with one router traversal and $D_{l}$ denote the propagation delay of a conventional one-pitch Mesh link. For a backbone link spanning s router pitches, its communication delay can be approximated as

(8) $$D_{backbone}=D_{r}+sD_{l}$$

while transmitting across the same physical distance through s conventional Mesh hops require

(9) $$D_{Mesh}=s\left(D_{r}+D_{l}\right)$$

Therefore, even when the wire delay of the backbone link is assumed to increase linearly with its physical length, the backbone traversal still eliminates the intermediate router traversal delays.

A similar relationship can be used to estimate communication energy. Let $E_{r}$ denote the energy associated with one router traversal and $E_{l}$ denote the energy required for transmission over one router-pitch link. The energy of a backbone traversal spanning s router pitches can be approximated as

(10) $$E_{backbone}=E_{r}+sE_{l}$$

whereas the corresponding s-hop Mesh path requires

(11) $$E_{Mesh}=s\left(E_{r}+E_{l}\right)$$

Thus, although the backbone link introduces a longer physical interconnect, it avoids the router-processing energy associated with intermediate hops. The above estimation is used only to explain the architectural trade-off between longer links and reduced router traversals. Detailed physical effects, including wire RC delay, repeater insertion, clocking overhead, and post-layout timing, remain implementation-dependent and are beyond the scope of the current architecture-level evaluation.

The evaluation metrics used in this work are defined as follows. The packet injection rate (PIR) represents the probability that a processing element generates a packet in each simulation cycle, and it is dimensionless. The average latency is measured as the average number of clock cycles from packet generation to successful reception. The normalized throughput is defined as the number of successfully transmitted flits divided by the simulation cycles and the number of processing elements, with the unit of flits/cycle/IP. The hop count represents the number of router-to-router transfers required for a packet to reach its destination. For HS-DAQ traffic, the injection interval represents the number of simulation cycles between two consecutive packet generations from an acquisition node, which is determined according to the data generation rate of the acquisition system and the configured packet payload.

### 5.1. Performance Evaluation Under General Communication Scenarios

#### 5.1.1. Results Under Different Injection Rates

Although synthetic patterns such as hotspot and transpose traffic are commonly used for general NoC evaluation, they do not represent the dominant communication behavior of HS-DAQ systems. Therefore, this work adopts random traffic for general scalability evaluation and application-derived HS-DAQ traffic for realistic workload validation.

To evaluate the network performance under different traffic injection conditions, the network size was fixed at 4 × 4 × 4, and the packet injection rate (PIR) was gradually increased from 0.01 to 0.09 in the simulator. To reduce the influence of stochastic traffic generation, each random traffic experiment was independently executed ten times with different random seeds. The reported latency results are presented as the average value with standard deviation.

As shown in Table 3, the standard deviation of the average latency remains relatively small under low and medium injection rates, indicating that the observed performance improvement is not caused by random fluctuations. Even under high injection rates where network congestion becomes significant, the variation among independent runs remains within an acceptable range.

Compared with latency, throughput and energy consumption exhibit much smaller variations because they are mainly determined by the overall packet generation rate and the fixed communication behavior of the network. Therefore, only the latency variation is explicitly reported.

The average of the results from 10 simulations was taken, and the corresponding results are shown in Figure 8. As shown in Figure 8a, under low injection rates, the overall network traffic load is relatively light, and the difference in average latency between the two architectures is small. As the injection rate increases, the network traffic load gradually rises and the network approaches congestion, resulting in a rapid increase in communication latency. Compared with the 3D Mesh NoC, the 3D AB-NoC exhibits lower average latency across all injection rates.

Figure 8b shows the comparison of network throughput between the two network architectures. Under low injection rates, no significant congestion is observed in the network. Since the throughput is mainly determined by the data injection rate, the two architectures exhibit nearly identical throughput. As the injection rate further increases, the network gradually enters a high-load operating state, and the throughput of the 3D AB-NoC becomes slightly higher than that of the 3D Mesh NoC. This indicates that the 3D AB-NoC can provide more efficient data transmission capability under high network communication loads.

Figure 8c shows a comparison of the total energy consumption results for the two network architectures at different injection rates. The results show that the total energy consumption of the 3D AB-NoC is consistently lower than that of the 3D Mesh NoC across all injection rates. As the injection rate increases, the difference in power consumption between the two architectures gradually becomes larger.

The above comparisons indicate that the performance differences between the two network architectures are relatively limited under low injection rates. As the injection rate increases, the performance advantages of the 3D AB-NoC gradually emerge. Across the evaluated injection rates, compared with the 3D Mesh NoC, the 3D AB-NoC reduces the average communication latency by approximately 2.23–19.80% and the total energy consumption by approximately 2.88–5.45%, while improving throughput by approximately 1.88–2.60%.

#### 5.1.2. Simulation Results Under Different Network Sizes

We then conducted comparative simulations of 3D AB-NoC and 3D Mesh NoC with different network sizes, under a fixed injection rate of PIR = 0.02. The average latency and corresponding standard deviation are summarized in Table 4. The standard deviations of both 3D AB-NoC and 3D Mesh remain relatively small across different network sizes, indicating that the performance comparison is stable under stochastic traffic conditions.

The results are shown in Figure 9. As shown in Figure 9a, the average latency of both architectures increases with network size. The 3D AB-NoC consistently exhibits lower average latency than the 3D Mesh NoC across all evaluated network sizes. As the network size increases, the 3D AB-NoC becomes more effective in reducing the average network latency and improving overall communication efficiency.

As shown in Figure 9b, under a fixed injection rate, neither network architecture exhibits a clear overall advantage in terms of throughput. This is mainly because network throughput is largely proportional to the data injection rate. When no network congestion occurs, the influence of topology on throughput is relatively limited. Therefore, under the same injection rate, the two architectures exhibit similar throughput performance. As shown in Figure 9c, the total energy consumption of the 3D AB-NoC is lower than that of the 3D Mesh NoC under all evaluated network sizes. Moreover, the difference in power consumption between the two architectures gradually increases as the network size expands.

These results demonstrate that, under a fixed injection rate, the 3D AB-NoC outperforms the 3D Mesh NoC in terms of average communication latency and power consumption as the network size increases. There is little difference between the two network architectures in terms of network throughput.

It should be noted that 3D AB-NoC is not intended as an equal-resource replacement of the conventional 3D Mesh. Instead, it is an application-oriented NoC architecture that introduces dedicated backbone resources to exploit the deterministic aggregation communication characteristics of HS-DAQ systems.

Therefore, the comparison focuses on whether the additional backbone resources can provide sufficient communication benefits under aggregation-dominated workloads. The hardware overhead introduced by the backbone subnet has been analyzed in Section 4.5.

### 5.2. Performance Evaluation Under HS-DAQ Communication Scenarios

To improve the reproducibility of the HS-DAQ traffic experiments, the node mapping configurations used in different network sizes are summarized in Table 5. In the simulation, acquisition nodes periodically generate packets and transmit them to predefined storage nodes according to the source-destination mapping. The same acquisition-storage communication pattern is maintained for different network dimensions, while the node coordinates are adjusted according to the network size.

#### 5.2.1. Results Under Different Data Injection Intervals

To evaluate the communication performance of the proposed 3D AB-NoC architecture in high-speed data acquisition scenarios, the network size was fixed at 6×6×4, while different data acquisition rates were emulated by adjusting the data injection interval of the acquisition nodes. The injection intervals were set to 24, 22, 20, 18, 16, and 14 clock cycles. The performance comparison between the 3D Mesh NoC and the 3D AB-NoC under different injection intervals is shown in Figure 10.

Figure 10a shows the average latency of the two network architectures under different data injection intervals. When the injection interval is relatively large, i.e., 24 and 22 clock cycles, the overall network load is light and the performance difference between the two architectures is small. As the injection interval decreases, the amount of aggregation traffic in the network increases, and the conventional 3D Mesh gradually experiences noticeable congestion. In contrast, the 3D AB-NoC, by introducing an aggregation backbone subnet, effectively reduces the average number of hops for packet transmission. Consequently, it is able to maintain a low average latency even at high data acquisition rates.

The network throughput of the two architectures under different data injection intervals is shown in Figure 10b. At relatively large injection intervals, the two architectures exhibit nearly identical throughput. As the injection interval decreases, the network gradually enters a high-load operating state, and the throughput gap between the two architectures becomes increasingly evident. When the injection interval is reduced to 14 clock cycles, the throughput of the 3D AB-NoC reaches 0.999 flits/cycle, whereas that of the 3D Mesh remains at only 0.773 flits/cycle, corresponding to an improvement of approximately 29.3%. The 3D AB-NoC can make more effective use of available network bandwidth under persistent aggregation traffic conditions.

Compared with average latency, maximum latency provides a more direct indication of data transmission performance under severe network congestion. Figure 10c shows the maximum communication latency of the two architectures under different data injection intervals. The maximum latency of the conventional 3D Mesh increases rapidly as the injection interval decreases. When the injection interval ranges from 20 to 16 clock cycles, the maximum latency increases from 305.2 cycles to 12,704 cycles, indicating that the network has entered a severely congested state. In comparison, the maximum latency of the 3D AB-NoC remains at approximately 25 cycles over the same injection-interval range and increases to only 124.2 cycles even under the minimum data injection interval.

The total energy consumption of the two network architectures under different data injection intervals is shown in Figure 10d. Across all evaluated injection intervals, the total energy consumption of the 3D AB-NoC is lower than that of the conventional 3D Mesh. When the injection interval ranges from 24 to 18 clock cycles, the total energy consumption of the 3D AB-NoC decreases by 6.3–12.9%. Even under the highest communication load conditions, the total energy consumption of the 3D AB-NoC remains slightly lower than that of the 3D Mesh NoC.

Overall, the results show that, in high-speed data acquisition communication scenarios, the 3D AB-NoC maintains lower communication latency, higher throughput, and lower power consumption than the conventional 3D Mesh NoC.

#### 5.2.2. Scalability Evaluation Under HS-DAQ Communication Scenarios

To further evaluate the scalability of the proposed architecture, the data injection interval was fixed at 24 clock cycles, corresponding to a condition under which no obvious network congestion occurred, and simulations were conducted for different network sizes. The results are shown in Figure 11.

The average communication latency of the two network architectures under different network sizes is shown in Figure 11a. As the network size increases, the average communication latency of both architectures gradually increases. Compared with the 3D Mesh NoC, the 3D AB-NoC consistently achieves lower average latency across all evaluated network sizes. Moreover, the performance advantage of the 3D AB-NoC becomes more pronounced as the network size increases, with the reduction in average latency increasing from 4.6% to 25.3%.

Figure 11b presents the throughput of the two network architectures under different network sizes. The throughput of both architectures remains approximately 0.583 flits/cycle, with almost no noticeable difference between them. Similar to the results under the random traffic injection mode, because the data injection interval is fixed at 24 clock cycles and the network remains uncongested, the system throughput is primarily determined by the data injection rate.

The maximum communication latency of the two architectures under different network sizes is shown in Figure 11c. Compared with the 3D Mesh NoC, the 3D AB-NoC maintains lower maximum communication latency across all evaluated network sizes, achieving a reduction of approximately 8.69–25.64%. This indicates that high-speed data acquisition systems can maintain favorable communication performance in 3D AB-NoC. Figure 11d shows the total energy consumption of the two architectures under different network sizes. The power consumption of the 3D AB-NoC is 10.92–30.82% lower than that of the 3D Mesh NoC.

#### 5.2.3. Sensitivity Analysis of Aggregation-Router Spacing

To investigate the influence of aggregation-router density on network performance, a sensitivity experiment was conducted by varying the backbone spacing s. The network size was fixed at 6×6×4, and the same HS-DAQ traffic configuration was used for all cases. The packet injection interval was set to 24 cycles, while the spacing parameter was varied as s=2,3,4. All other simulation parameters, including the acquisition-node locations, storage-node locations, packet size, buffer depth, simulation duration, and source-destination relationships, were kept unchanged.

According to the placement rule defined in Section 4.1, s=2 results in 36 aggregation routers and 48 backbone links, whereas both s=3 and s=4 contain 16 aggregation routers and 16 backbone links in the 6×6×4 network. Although s=3 and s=4 have the same numbers of aggregation routers and backbone links, their aggregation-router locations and backbone-link spans are different. Therefore, the spacing parameter affects not only backbone density but also the alignment between the backbone subnet and the application traffic mapping. The experimental results are shown in Table 6.

The results show that the performance of 3D AB-NoC is sensitive to the aggregation-router spacing rather than varying monotonically with backbone density. When s=2, the average latency is 20.4964 cycles. Increasing the spacing to s=3 slightly increases the average latency to 21.3703 cycles, whereas s=4 reduces it substantially to 17.0053 cycles. The throughput remains relatively stable for all three configurations, ranging from 0.583056 to 0.583761 flit/cycle. A similar trend is observed for energy consumption: the (*s* = 4) configuration achieves the lowest total energy of 0.00756117 J.

These results indicate that increasing the number of aggregation routers does not necessarily lead to better performance. The spacing parameter determines both backbone density and its spatial correspondence with the dominant traffic paths. Therefore, the preferred spacing depends on the network dimensions and application mapping.

It should be noted that the above sensitivity study is intended to illustrate the influence of the backbone spacing on performance and structural overhead, rather than to derive a universally optimal spacing. The preferred value of (s) depends jointly on the network dimensions, the locations of acquisition and storage nodes, and the dominant aggregation paths. In the main experiments of this work, s=2 is adopted as a fixed baseline configuration to provide a consistent backbone deployment across the evaluated workloads.

Based on the above simulation results, it can be seen that, under the same data acquisition rate and without obvious network congestion, the 3D AB-NoC achieves lower average latency, maximum latency, and total energy consumption across different network sizes. These results demonstrate that the proposed aggregation backbone subnet can effectively reduce long-distance communication overhead in large-scale HS-DAQ systems. Consequently, the 3D AB-NoC improves system scalability and communication efficiency, providing a more efficient on-chip interconnection solution for large-scale high-speed data acquisition systems.

### 5.3. Performance Evaluation Based on an Application-Derived HS-DAQ Workload

To further evaluate the proposed 3D AB-NoC under a workload derived from a practical high-speed data acquisition system, the parameters of the multi-channel acquisition system for high-frequency ground-wave radar (HFGWR) reported in [22] were adopted. The referenced system employs ADS42LB69 ADC devices for high-speed signal acquisition. In the proposed NoC-level evaluation, each ADC is abstracted as an independent acquisition source node. Therefore, eight ADC devices are modeled as eight acquisition nodes to represent the continuous data generation characteristics of the HS-DAQ system. The acquired data are transmitted from acquisition nodes to storage nodes through the NoC according to the predefined communication mapping.

The eight acquisition ADCs are mapped to eight acquisition nodes, while two storage nodes are used to represent the shared buffering resources of the HS-DAQ architecture. According to the maximum data output capability of the ADS42LB69 ADC, the data rate of each ADC is configured as 3.125 Gbps. In this experiment, this value is adopted as the input data rate of each acquisition node to model the high-speed data stream in the HS-DAQ system.

In the Access Noxim model, the NoC operates at 1 GHz and the flit width is 32 bits. A fixed packet length of five flits was used for this experiment. Each packet consists of one head flit, three data flits, and one tail flit. The three data flits correspond to 96 bits of acquisition data. Therefore, the packet generation interval can be obtained as

(12) $$T_{packet}=L_{data}/R_{ADC}$$

where $L_{data}$ represents the valid data bits of a packet within the NoC, and $R_{ADC}$ denotes the data rate of a single ADC inputting into the NoC, which is 3.125 Gbps. The calculated packet generation interval is approximately 31 ns. With a NoC clock period of 1 ns, the corresponding packet injection interval is

(13) $$I_{inj}=T_{packet}/T_{clk}$$

where $T_{clk}$ is the NoC clock period. Thus, the application-derived workload uses a fixed packet size of five flits and an injection interval of 31 cycles. All acquisition nodes generate packets periodically and synchronously.

A 4×4×3 NoC was used for this experiment. The acquisition nodes were located at node IDs 24, 28, 40, 44, 26, 30, 42, and 46, while the two storage nodes were located at node IDs 0 and 2. The first four acquisition nodes communicate with the first storage node, and the last four acquisition nodes communicate with the second storage node. The backbone spacing of 3D AB-NoC was set to 2. Except for the topology and routing mechanism, all workload parameters, including packet generation times, packet sizes, source nodes, and destination nodes, were kept identical for the 3D Mesh and 3D AB-NoC.

Table 7 presents the performance results under the application-derived HFGWR workload. Both networks successfully delivered the same number of packets and flits, indicating that the offered workloads were identical. Compared to the 3D Mesh NoC, the 3D AB-NoC achieves an 11.8% reduction in average network latency, a 7.7% reduction in maximum communication latency, and a 21% reduction in total network power consumption. The global throughput of both architectures was 0.750067 flit/cycle, indicating that both networks were able to sustain the application-derived traffic load without throughput degradation.

These results indicate that under a workload derived from the documented parameters of a practical multi-channel radar acquisition system, the proposed architecture still achieves lower communication latency and network energy while maintaining the same throughput.

## 6. Conclusions

To address the mismatch between the data transmission characteristics of time-interleaved high-speed data acquisition systems and conventional NoC architectures, this paper proposes a 3D aggregation backbone network-on-chip architecture. While preserving the regular structure of the 3D Mesh, the proposed architecture constructs a coarse-grained aggregation backbone subnet through periodically deployed aggregation routers and additional backbone links. Meanwhile, a Backbone-Boosted XYZ routing algorithm is proposed to enable long-distance aggregation traffic to be efficiently forwarded through the backbone subnet.

Experimental results demonstrate the effectiveness of the proposed architecture. Under the HS-DAQ traffic scenario, compared with the conventional 3D Mesh, the proposed 3D AB-NoC reduces the average latency by approximately 44.1% and improves the throughput by 29.3% under high-load conditions, while maintaining lower energy consumption. Furthermore, based on the application-derived HFGWR workload, the proposed architecture achieves a reduction of approximately 11.8% in average latency and 21.0% in energy consumption compared with the conventional 3D Mesh. These results verify that the proposed aggregation-oriented topology and routing strategy can effectively improve communication efficiency for HS-DAQ systems. Future work will further focus on determining the optimal spacing between aggregation nodes for arbitrary network scales and application mappings, as well as investigating corresponding analytical or optimization methods.

## Figures and Tables

**Figure 1:**
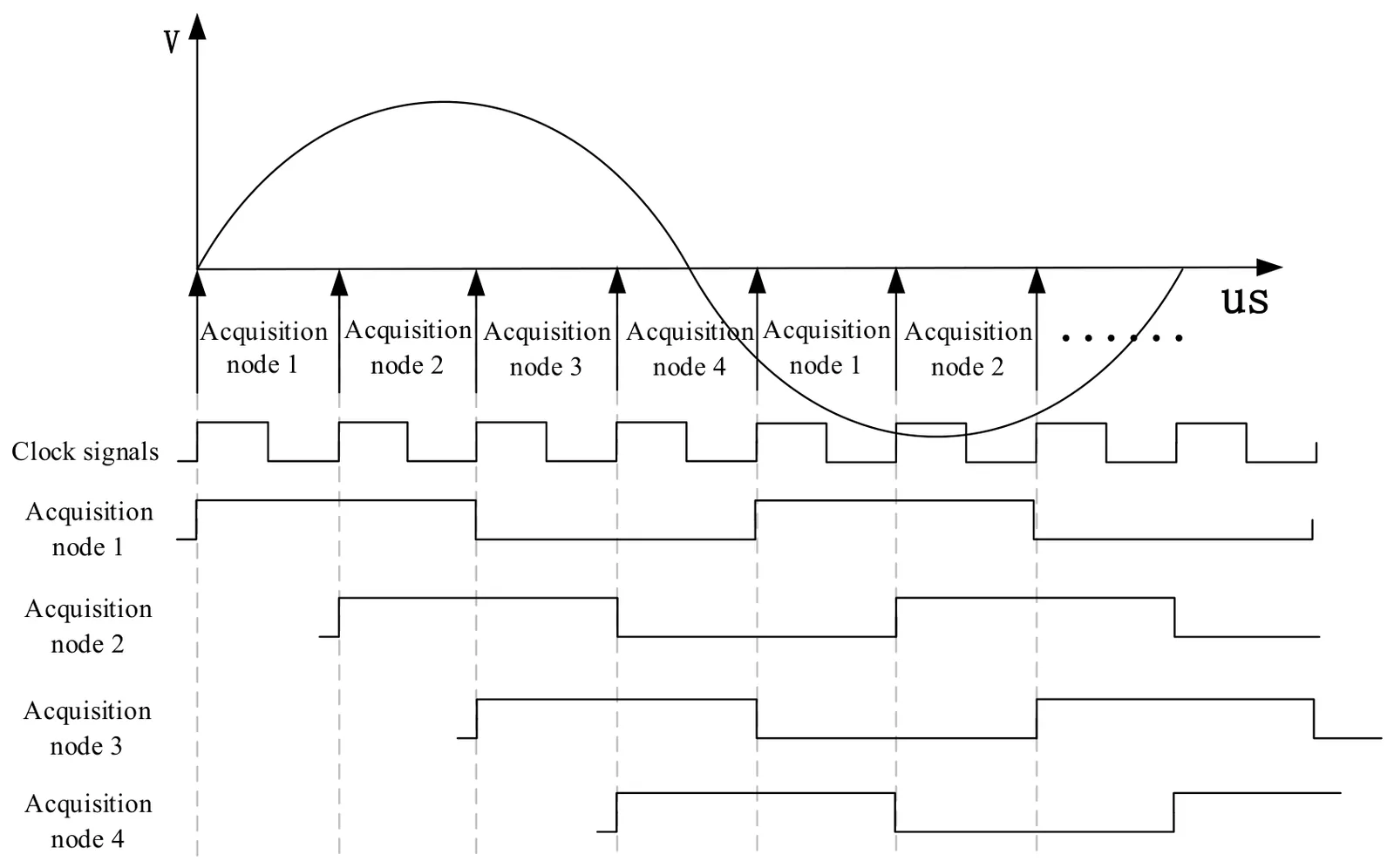
Time interleaved sampling timing of the four acquisition nodes. The ellipsis indicates the periodic continuation of the sampling sequence.

**Figure 2:**
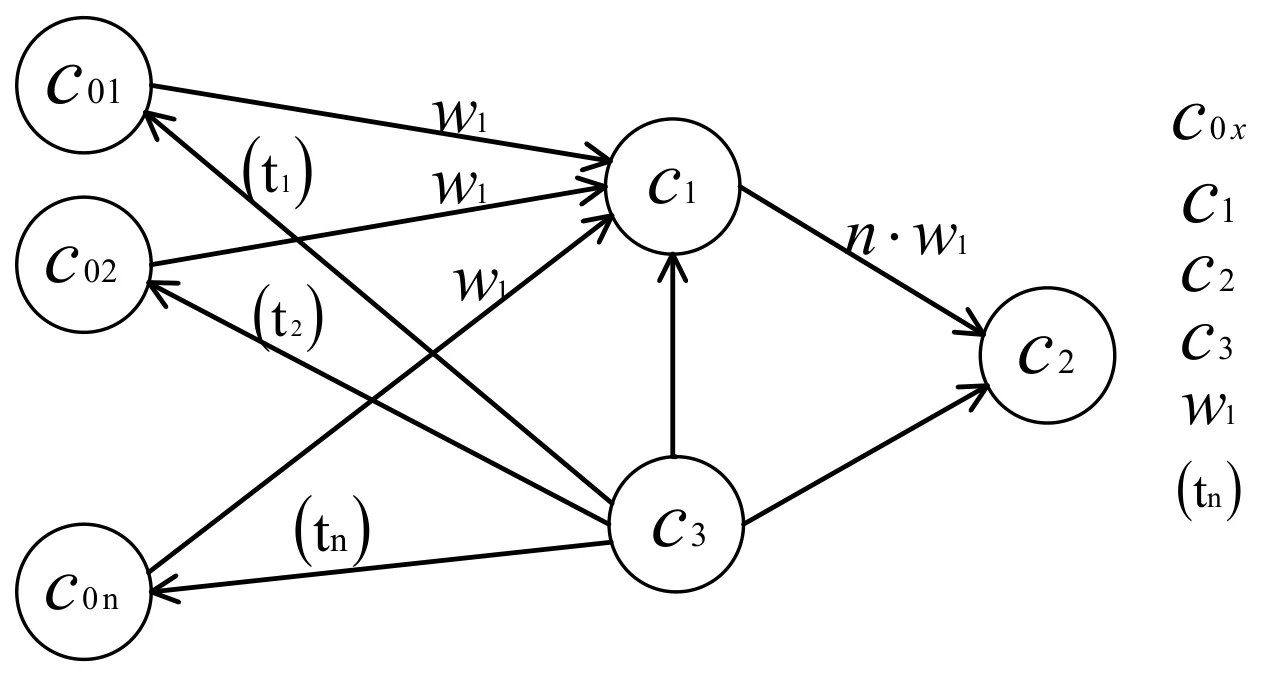
Task diagram of the data acquisition system. The ellipsis indicates additional acquisition channels that are omitted for clarity.

**Figure 3:**
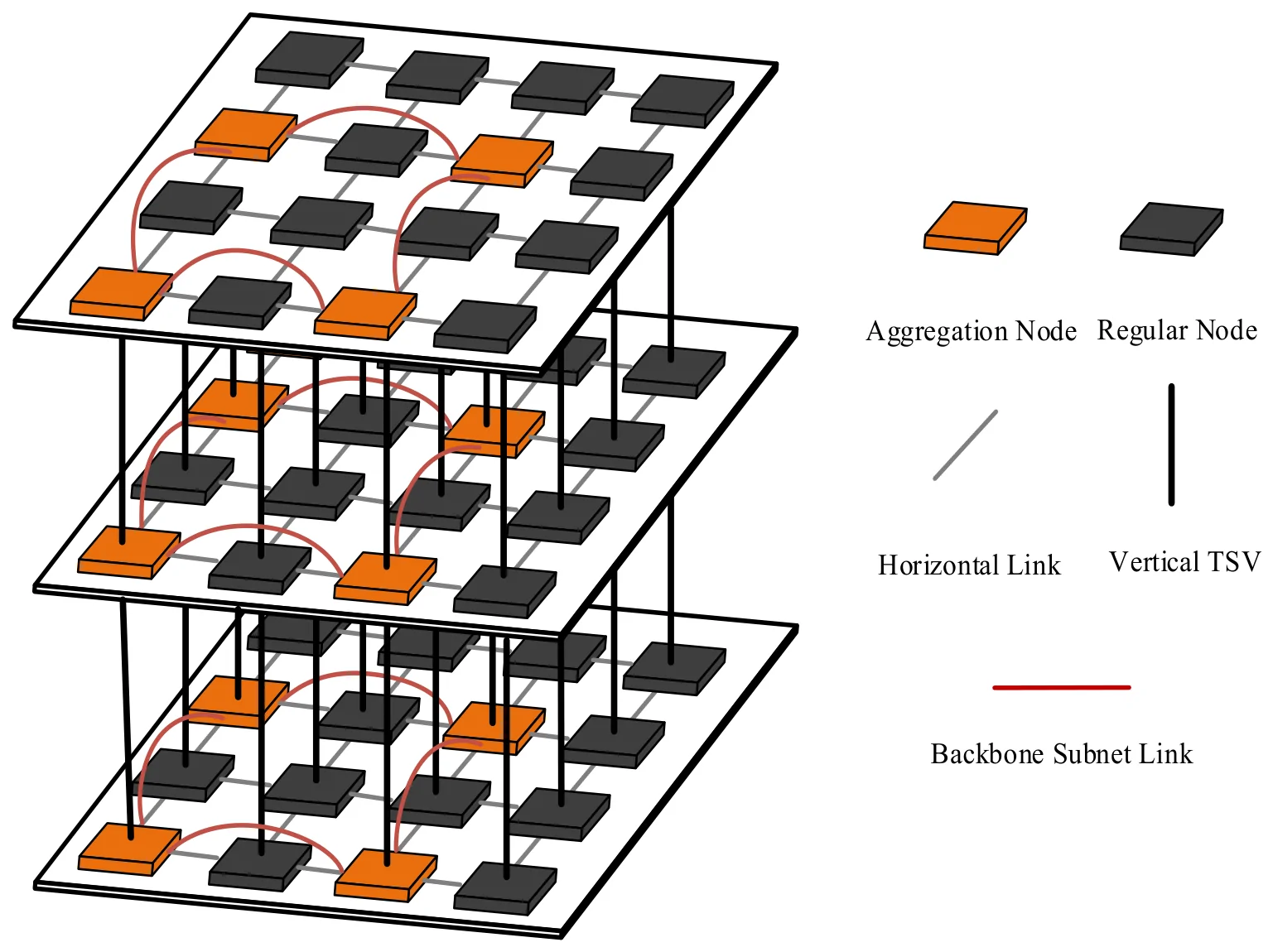
The 4 × 4 × 3 3D AB-NoC architecture.

**Figure 4:**
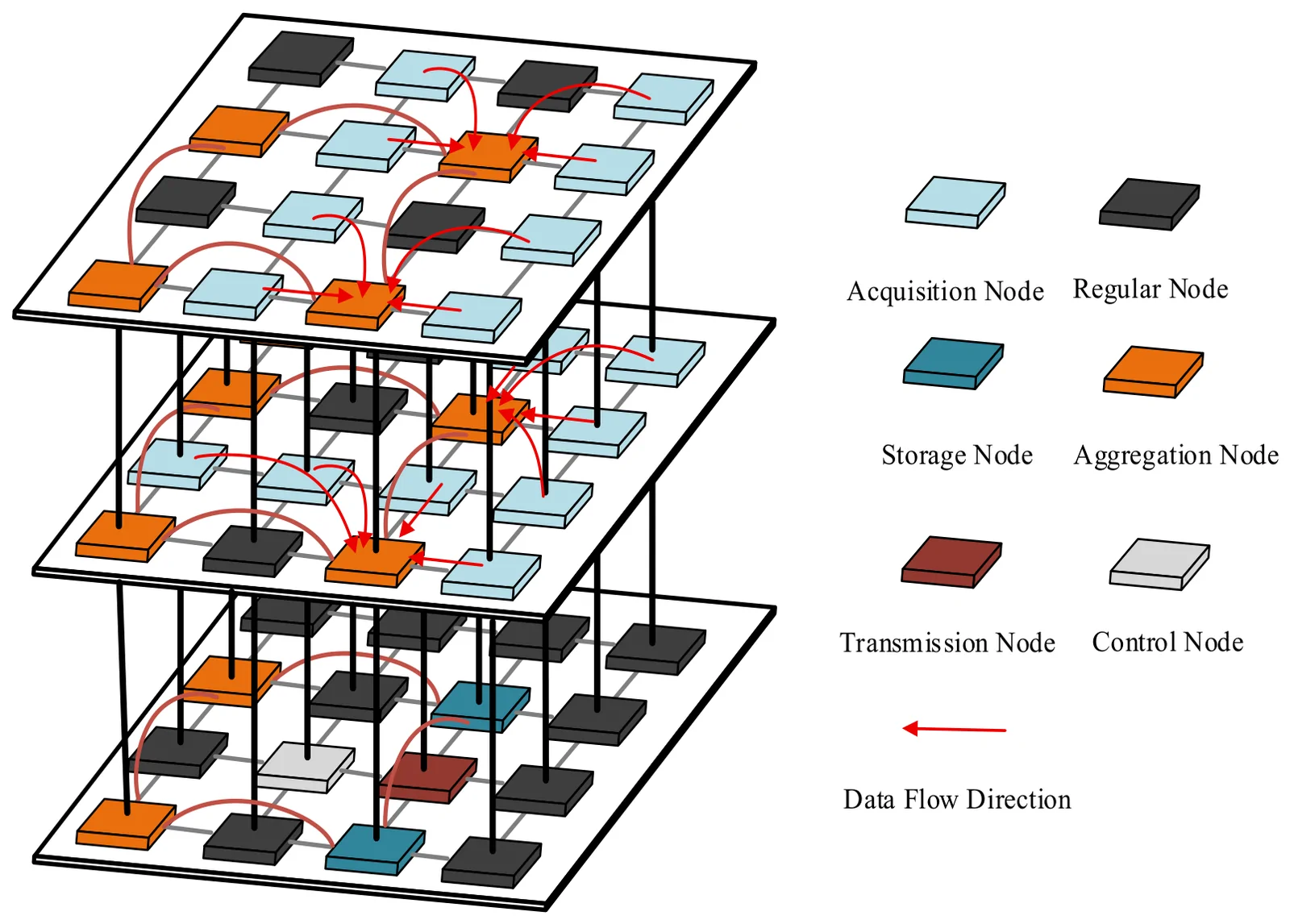
HS-DAQ system model based on 3D AB-NoC.

**Figure 5:**
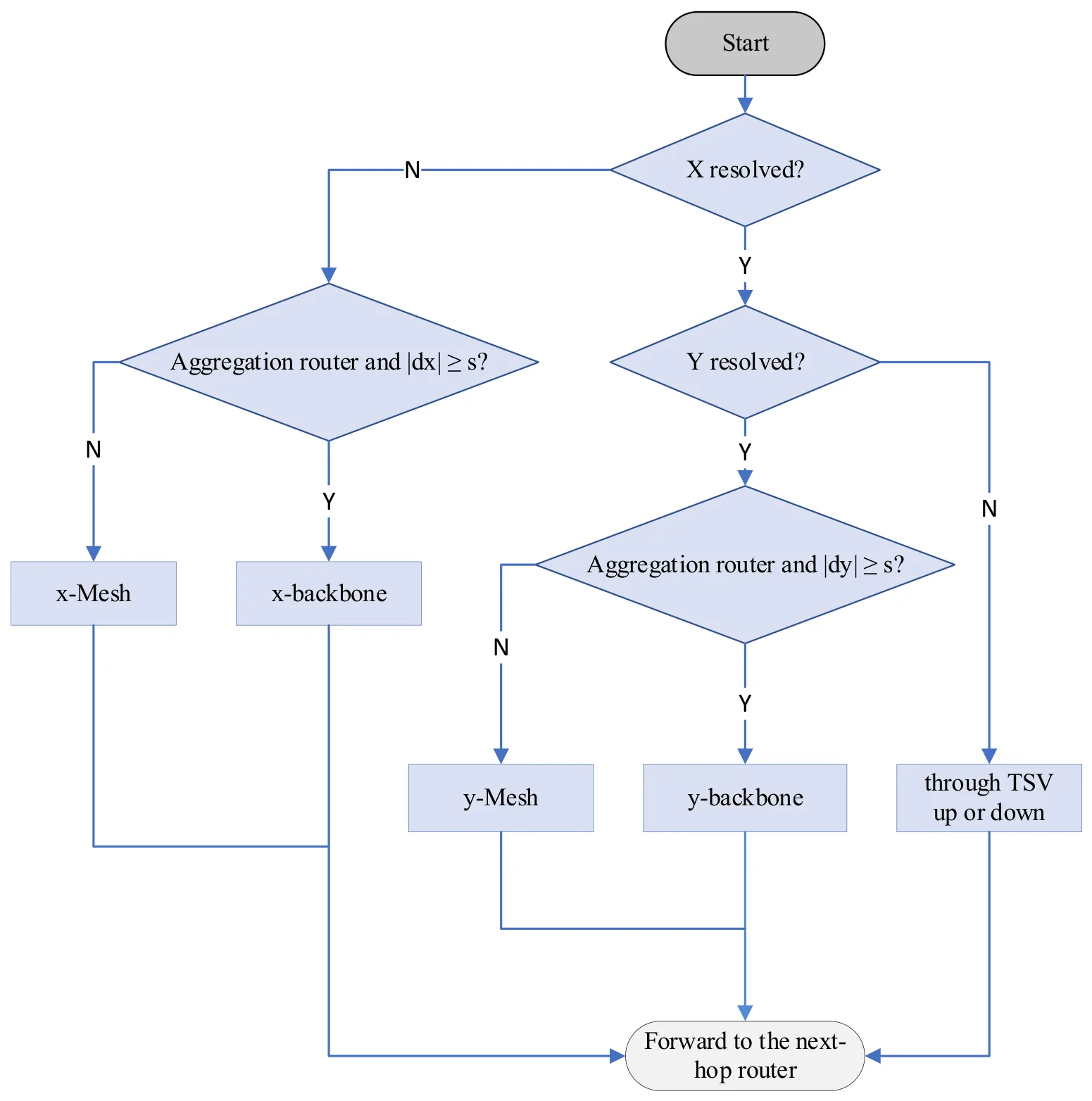
Flowchart of the Backbone-Boosted XYZ routing algorithm.

**Figure 6:**
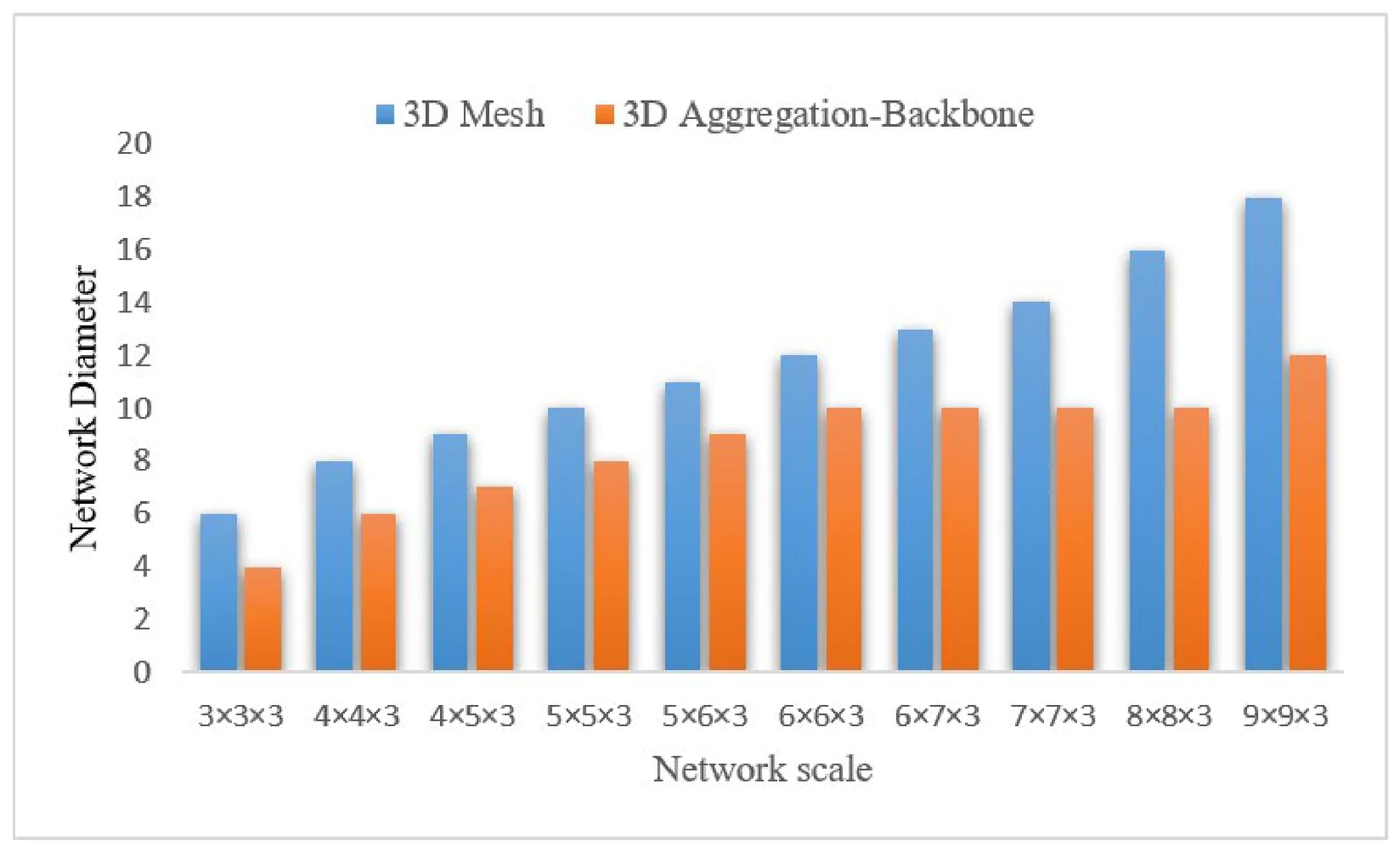
Comparison of network diameter under different network sizes.

**Figure 7:**
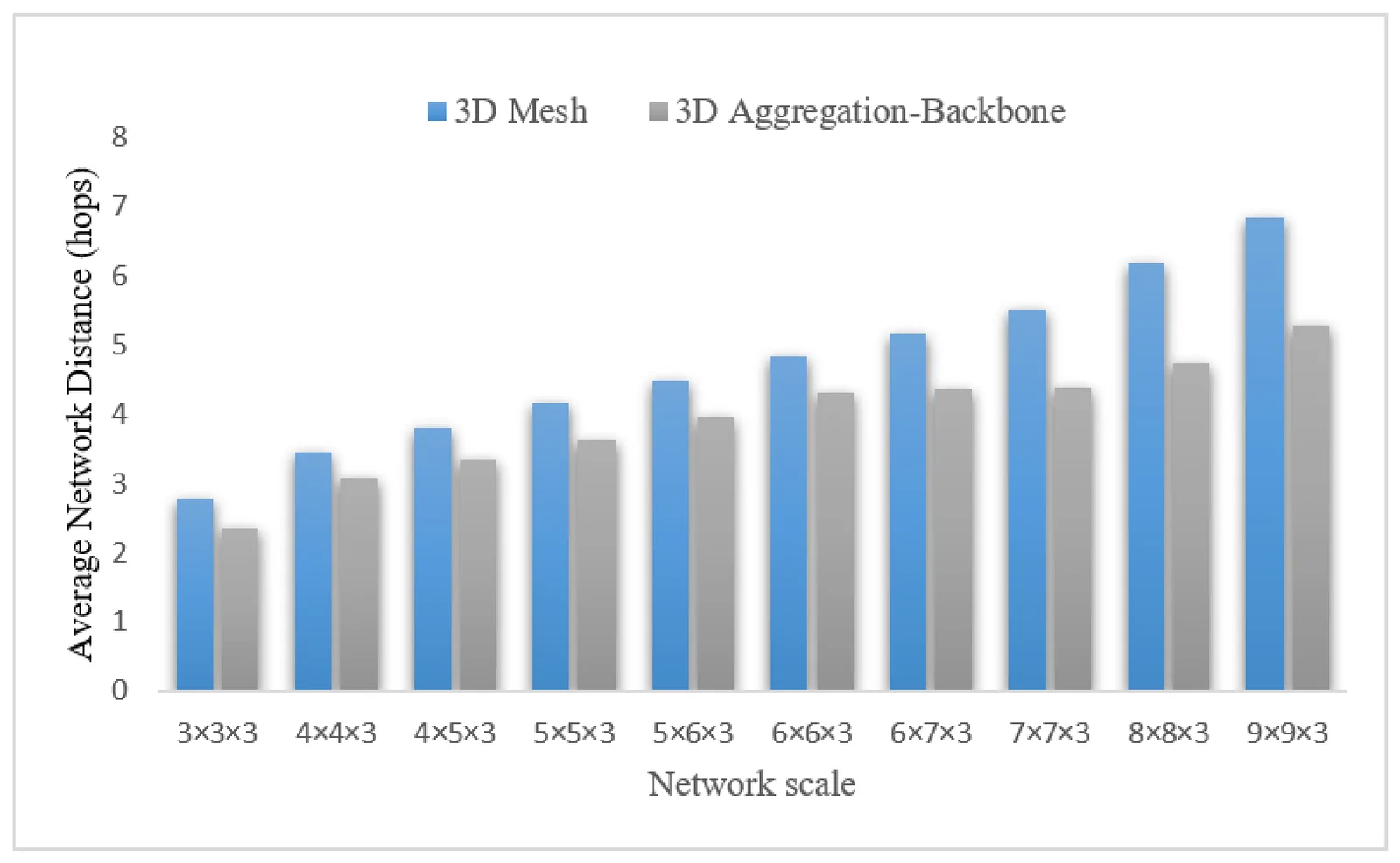
Comparison of average network distance under different network sizes.

**Figure 8:**
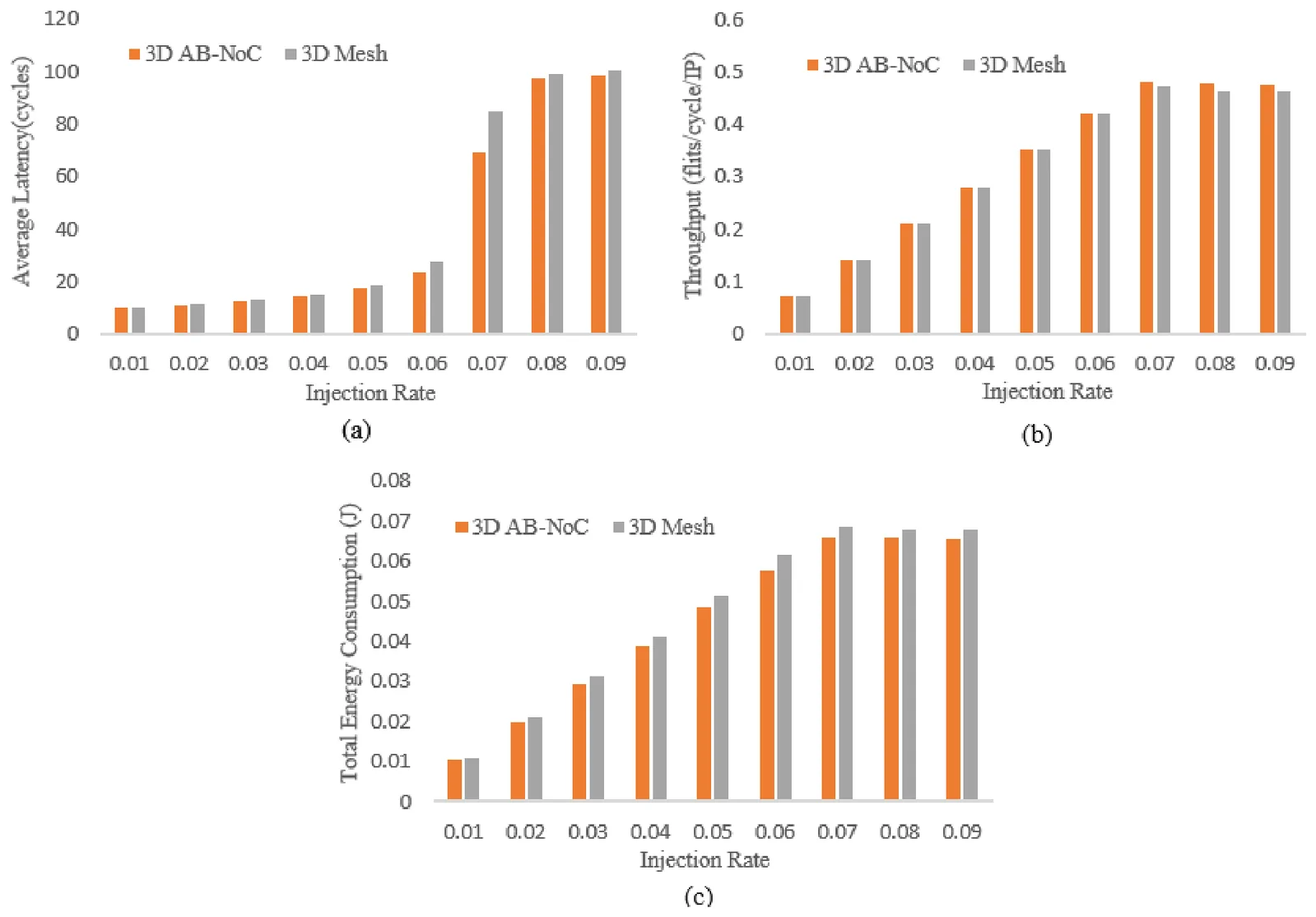
Performance comparison under different injection rates in general communication scenarios: (**a**) average latency. (**b**) Throughput. (**c**) Total energy consumption.

**Figure 9:**
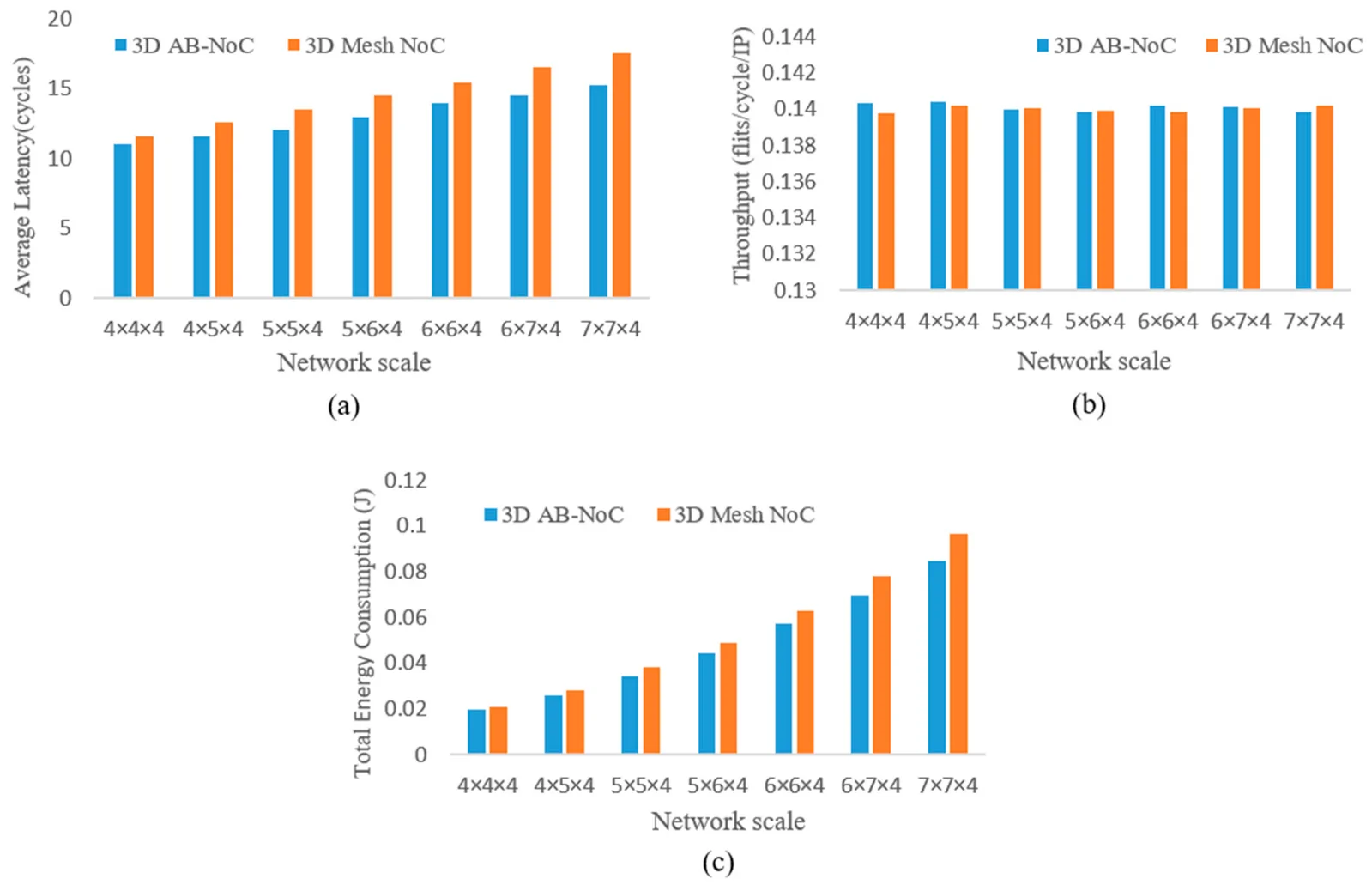
Performance comparison under different network sizes in general communication scenarios: (**a**) average latency. (**b**) Throughput. (**c**) Total energy consumption.

**Figure 10:**
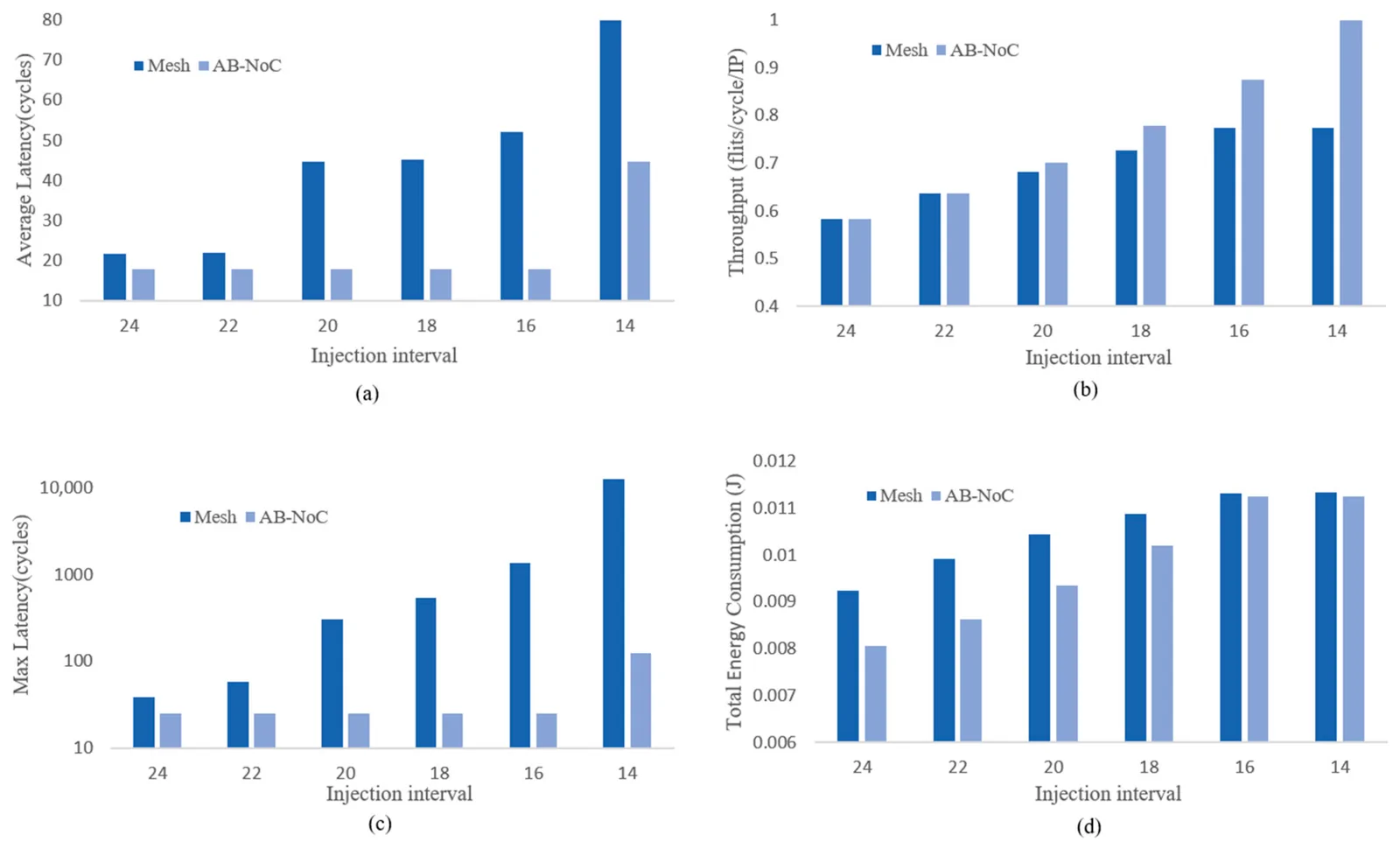
Performance comparison of 3D Mesh NoC and 3D AB-NoC under different data injection intervals in HS-DAQ communication scenarios: (**a**) Average latency. (**b**) Throughput. (**c**) Maximum communication latency. (**d**) Total energy consumption.

**Figure 11:**
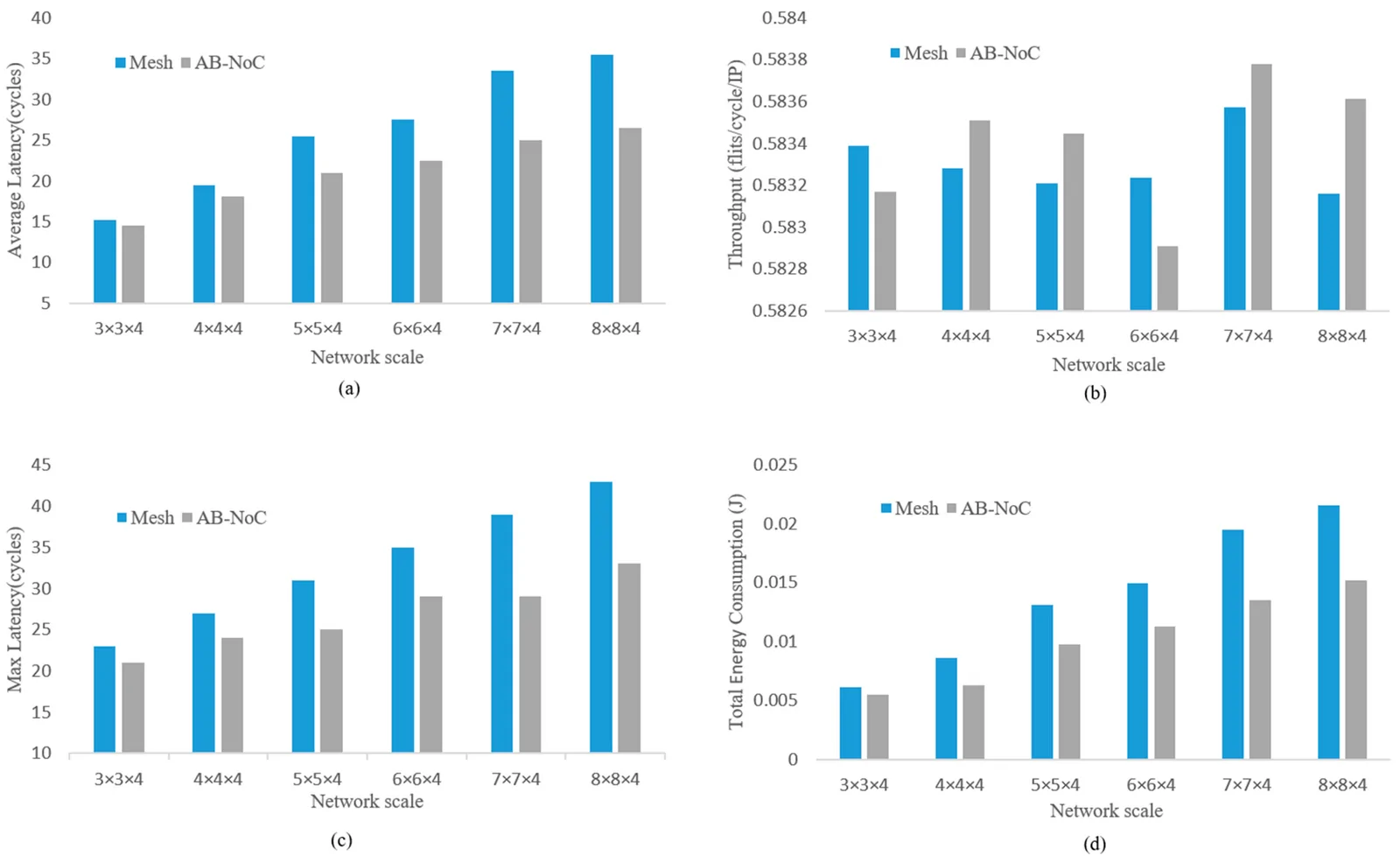
Performance comparison of 3D Mesh NoC and 3D AB-NoC under different network sizes in HS-DAQ communication scenarios: (**a**) Average latency. (**b**) Throughput. (**c**) Maximum communication latency. (**d**) Total energy consumption.

**Table 1:** Comparison of existing NoC architectures and the proposed 3D AB-NoC.

Architecture	Additional Connectivity	Router Modification	Routing Mechanism	Target Traffic
CMesh	Cluster-based links	Cluster router	Cluster routing	General applications
DiamondMesh	Diagonal links	Additional diagonal ports	XY-based routing	General applications
Small-world NoC	Sparse long-range links	Additional long links	Adaptive routing	General applications
Hierarchical NoC	Multi-level links	Hierarchical routers	Hierarchical routing	Large-scale many-core systems
3D PC-NoC	Optimized TSV links	Vertical-link optimization	Elevator/scheduling strategies	3D NoC communication
3D AB-NoC (this work)	Sparse aggregation backbone links	Aggregation routers with backbone ports	BB-XYZ deterministic routing	HS-DAQ many-to-few aggregation traffic

**Table 2:** Simulation parameter settings.

Network Parameter	Parameter Setting
Topology	3D Mesh NoC, 3D AB-NoC
Routing Algorithm	XYZ routing, BB-XYZ routing
Buffer depth	8 flits
Packet size	6 flits
Flit width	32 bits
Traffic patterns	Random traffic and HS-DAQ traffic
Flow control	Wormhole switching
Router energy model	Noxim default router/link model
Simulation Time	100,000 clock cycles
Warm-up Time	1000 clock cycles

**Table 3:** Average latency comparison under different injection rates.

Injection Rate	3D AB-NoC (Cycles)	3D Mesh (Cycles)
0.01	10.040 ± 0.021	10.514 ± 0.020
0.02	11.092 ± 0.033	11.655 ± 0.032
0.03	12.491 ± 0.021	13.167 ± 0.025
0.04	14.391 ± 0.048	15.302 ± 0.051
0.05	17.303 ± 0.136	18.824 ± 0.092
0.06	23.618 ± 0.262	27.414 ± 0.637
0.07	69.360 ± 2.224	84.978 ± 1.918
0.08	97.012 ± 0.635	99.253 ± 0.530
0.09	98.291 ± 0.469	100.553 ± 0.297

**Table 4:** Average latency comparison under different network sizes.

Network Size	3D AB-NoC (Cycles)	3D Mesh (Cycles)
4 × 4 × 4	11.0000 ± 0.0199	11.5786 ± 0.2255
4 × 5 × 4	11.5514 ± 0.0159	12.5602 ± 0.0423
5 × 5 × 4	12.0719 ± 0.0191	13.4986 ± 0.0262
5 × 6 × 4	12.9792 ± 0.0232	14.4680 ± 0.0232
6 × 6 × 4	13.9260 ± 0.0244	15.4385 ± 0.0303
6 × 7 × 4	14.5245 ± 0.0231	16.4836 ± 0.0261
7 × 7 × 4	15.1158 ± 0.0217	17.5454 ± 0.0297

**Table 5:** Node mapping configuration for the HS-DAQ traffic model.

Network Size	Acquisition Nodes	Storage Nodes	Source-Destination Mapping
3 × 3 × 4	35,34,33,32,29,28,27,26	0,2,6,8	35, 34 to 0; 33, 32 to 2; 29, 28 to 6; 27, 26 to 8
4 × 4 × 4	63,62,60,61,51,50,48,49	0,2,8,10	63, 62 to 0; 60, 61 to 2; 51, 50 to 8; 48, 49 to 10
5 × 5 × 4	99,98,95,96,79,78,75,76	0,4,20,24	99, 98 to 0; 95, 96 to 4; 79, 78 to 20; 75, 76 to 24
6 × 6 × 4	143,142,138,139,113,112,108,109	0,4,24,28	143, 142 to 0; 138, 139 to 4; 113, 112 to 24; 108, 109 to 28
7 × 7 × 4	195,194,189,190,153,152,147,148	0,6,42,48	195, 194 to 0; 189, 190 to 6; 153, 152 to 42; 147, 148 to 48
8 × 8 × 4	255,254,248,249,199,198,192,193	0,6,48,54	255, 254 to 0; 248, 249 to 6; 199, 198 to 48; 192, 193 to 54

**Table 6:** Performance and structural characteristics under different backbone spacings.

Spacing	Aggregation Routers	Backbone Links	Avg. Latency	Throughput	Total Energy
2	36	48	20.4964	0.583056	0.00989396
3	26	16	21.3703	0.583278	0.0102311
4	26	16	17.0053	0.583761	0.00756117

**Table 7:** Performance comparison under the application-derived HFGWR workload.

Metric	3D Mesh	3D AB-NoC
Average latency	16.9995	14.9995
Maximum latency	26	24
Throughput	0.750067	0.750067
Total energy	0.00409223	0.00323114

## Data Availability

The original contributions presented in this study are included in the article/Supplementary Materials. Further inquiries can be directed to the corresponding author.

## Supplementary Materials

The following supporting information can be downloaded at: https://www.mdpi.com/article/10.3390/mi17101119/s1. The supplementary materials include: Comparison in high-speed data acquisition traffic injection; Comparison of structural characteristics; Comparison under different architectures (random traffic injection); Comparison under different injection rates (random traffic injection).
